# Non-Rigid Structure Estimation in Trajectory Space from Monocular Vision

**DOI:** 10.3390/s151025730

**Published:** 2015-10-12

**Authors:** Yaming Wang, Lingling Tong, Mingfeng Jiang, Junbao Zheng

**Affiliations:** School of Information Science and Technology, Zhejiang Sci-Tech University, Hangzhou 310018, China; E-Mails: ywang@zstu.edu.cn (Y.W.); m.jiang@zstu.edu.cn (M.J.); zhengjunbao@zstu.edu.cn (J.Z.)

**Keywords:** non-rigid structure estimation, monocular vision, trace minimization constraint, rank minimization, APG algorithm

## Abstract

In this paper, the problem of non-rigid structure estimation in trajectory space from monocular vision is investigated. Similar to the Point Trajectory Approach (PTA), based on characteristic points’ trajectories described by a predefined Discrete Cosine Transform (DCT) basis, the structure matrix was also calculated by using a factorization method. To further optimize the non-rigid structure estimation from monocular vision, the rank minimization problem about structure matrix is proposed to implement the non-rigid structure estimation by introducing the basic low-rank condition. Moreover, the Accelerated Proximal Gradient (APG) algorithm is proposed to solve the rank minimization problem, and the initial structure matrix calculated by the PTA method is optimized. The APG algorithm can converge to efficient solutions quickly and lessen the reconstruction error obviously. The reconstruction results of real image sequences indicate that the proposed approach runs reliably, and effectively improves the accuracy of non-rigid structure estimation from monocular vision.

## 1. Introduction

Recently, non-rigid structure estimation from monocular vision, which can recover the time varying 3D coordinates of points on a non-rigid object from their 2D places in a video sequence, has become a popular research topic. Generally, two major methods, *i.e.*, the trajectory basis method and shape basis method, are often used to solve non-rigid structure estimation problems. The factorization method was first proposed to recover rigid structure by Tomasi and Kanade [1], and the method was extended to solve the non-rigid structure problem in the seminal paper by Bregler *et al.* [2]. The core idea is that shapes observed from motion can be represented by the linear combination of a compact set of basis shapes. Each instantaneous structure, such as a running person, can be expressed as a point in the linear space of shapes spanned by the shape basis. A large number of methods have subsequently been developed [3,4,5], which promoted improved performances of shape basis. However, the shape basis has some limitations since it has a specific nature and can’t generally apply to all non-rigid bodies. The shape basis of a dancer moving, for example, cannot be recycled to compactly represent a person running. So, as an alternative to a shape space, Akhter *et al.* [6,7] proposed to represent the time-varying structure of a non-rigid object by using a linear combination of a set of basis trajectories, which was called Point Trajectory Approach (PTA). The primary advantage of PTA was that the trajectory basis can be predefined to be close to many real trajectories, which resulted in a significant reduction in unknowns, and corresponding stability improvement in estimation. Zhu *et al.* [8] pointed out that the importance of selecting the number of trajectory basis, rather than the more bases used the better, which varied from different models. On this basis, Gotardo and Matinez [9] combined the shape method and trajectory method, which can further improve the reconstruction performance. Recently, Rehan *et al.* [10] proposed a novel constraint in the form of local rigidity, which gave stable results in challenging realistic scenarios with small camera motions and shorter sequences. Minsk *et al.* [11] introduced new constraints that were more effective for non-rigid structure estimation, which constrained the motion parameters so that the 3D shapes were most closely aligned to each other, making the rank constraints unnecessary. Then they proposed a new probabilistic model in [12], which incorporated the smoothness constraint without requiring any prior knowledge. This approach regarded the sequence of 3D shapes as a simple stationary Markov process with Procrustes alignment, whose parameters were learned during the fitting process. Antonio *et al.* [13] proposed an online solution to estimate non- rigid structure, which modeled non-rigid deformations as a linear combination of some mode shapes obtained using modal analysis from continuum mechanics. However, the underlying principle behind most approaches was to model deformations using a low-rank shape [2,9,14,15], and it improved the accuracy of the non-rigid structure estimation.

In order to further improve the accuracy of non-rigid structure estimation, low rank condition of structure matrix is also investigated in this paper, and the APG algorithm is proposed to optimize the structure matrix, which can quickly converge to an efficient solution. Many trajectory bases can be used to recover the structure [16], such as the Discrete Cosine Transform (DCT) basis, Walsh Hadamard Transform (WHT) basis and Discrete Wavelet Transform (DWT) basis. In this paper, the predefined DCT basis is introduced to recover the motion and structure of the non-rigid object. For the 2D signal of M×N sample points, the DCT formula can be defined as follows:

(1) $$y(k,d)=u(k)\sum_{n=1}^{N}u(n,d)\cos\frac{\text{π}(2N-1)(k-1)}{2N}$$

where k=1,2,…,M and μ(k) is the coefficient:

(2) $$u(k)=\left\{ \begin{array}{c} \frac{1}{\sqrt{M}},k=1\\ \sqrt{\frac{2}{M}},2\leq k\leq M \end{array}\right.$$

In the paper, the APG method is proposed to solve the problem of non-rigid structure estimation. A new constraint, called trace minimization constraint of the rectification matrix, is introduced to narrow the solution space and improve the computiational speed of our algorithm. The proposed method can effectively estimate both 3D structures of non-rigid objects and the camera motion. The experimental results on real image sequences indicate that the proposed approach effectively improves the accuracy of non-rigid structure estimation from monocular vision.

This paper is organized as follows: the problems are formally described in Section 2 before briefly introducing how to get the initial structure matrix S by using PTA method in Section 3. In Section 4, the APG algorithm is introduced to optimize S. Experimental results are presented in Section 5. Finally, a summary and future works are discussed.

## 2. The Problem of Non-Rigid Structure Estimation

In fact, 3D reconstruction of non-rigid motion from monocular vision is equivalent to the decomposition of the measurement matrix *W*, that is decomposing the W into the rotation matrix R of camera and the structure matrix S of the non-rigid object. This problem can be simplified to estimate the rectification matrix *Q*. The PTA method is implemented to estimate the corresponding unknown parameters by a series of constraints, and to recover the structure S of the non-rigid object. Then, the APG algorithm is used to reconstruct the structure matrix S which is calculated by PTA, and can further improve the accuracy of the non-rigid structure estimation from monocular vision.

After feature point correspondence, the measured 2D trajectories can be included in the measurement matrix *W*, containing the location of *N* image points across *M* frames:

(3) $$W=\left[\begin{array}{c} X\\ Y \end{array}\right]=\left[\begin{array}{ccc} x_{11} & \cdots & x_{1N}\\ y_{11} & \cdots & y_{1N}\\ \vdots & \vdots & \vdots \\ x_{M1} & \cdots & x_{MN}\\ y_{M1} & \cdots & y_{MN} \end{array}\right]$$

The measurement matrix *W* can be decomposed as W=RS, where R is a 2M×3M matrix, $R_{i}(i=1,2,\cdots M)$ is an orthogonal projection matrix:

(4) $$R=\left[\begin{array}{ccc} R_{1} & & \\ & \ddots & \\ & & R_{M} \end{array}\right]$$

The structure matrix S is a 3M×N matrix, and the structure at a time instant t can be represented as follows:

(5) $$S=\left[\begin{array}{ccc} X_{t1} & \cdots & X_{tN}\\ Y_{t1} & \cdots & Y_{tN}\\ Z_{t1} & \cdots & Z_{tN} \end{array}\right]$$

## 3. The Calculation of Matrix *S* Using PTA

The structure matrix *S* can be decomposed into the trajectory basis matrix Θ and the coefficient matrix A, $S_{3M\times N}={\text{Θ}}_{3M\times 3K}A_{3K\times N}$. Defining the equation Λ=RΘ, the elements of the matrix Λ are as follows:

(6) $$\Lambda =R\text{Θ}=\left(\begin{array}{ccc} r_{1}^{1}{\text{θ}}_{1}^{T} & r_{2}^{1}{\text{θ}}_{1}^{T} & r_{3}^{1}{\text{θ}}_{1}^{T}\\ r_{4}^{1}{\text{θ}}_{1}^{T} & r_{5}^{1}{\text{θ}}_{1}^{T} & r_{6}^{1}{\text{θ}}_{1}^{T}\\ & \vdots & \\ r_{1}^{F}{\text{θ}}_{1}^{T} & r_{2}^{F}{\text{θ}}_{1}^{T} & r_{3}^{F}{\text{θ}}_{1}^{T}\\ r_{4}^{F}{\text{θ}}_{1}^{T} & r_{5}^{F}{\text{θ}}_{1}^{T} & r_{6}^{F}{\text{θ}}_{1}^{T} \end{array}\right)$$

where:

(7) $$\text{Θ}={\left[\begin{array}{ccc} {\text{θ}}_{1}^{T} & & \\ & {\text{θ}}_{1}^{T} & \\ & & {\text{θ}}_{1}^{T}\\ & \vdots & \\ {\text{θ}}_{M}^{T} & & \\ & {\text{θ}}_{M}^{T} & \\ & & {\text{θ}}_{M}^{T} \end{array}\right]}_{3M\times 3K}$$

*K* is the size of the DCT basis. If *K* is chosen too small, the trajectory is poorly represented, but if it is chosen too large, the system is ill-conditioned and the reconstruction error becomes unlimited, so how to choose a suitable K is very important.

According to the above mentioned, the measurement matrix *W* is decomposed as:

(8) W=RS=RΘΑ=ΛΑ

Factorize W with the Singular Value Decomposition (SVD) method:

(9) $$W=\overset{\wedge }{\Lambda }\overset{\wedge }{\text{Α}}$$

However, the matrices $\overset{\wedge }{\text{Λ}}$ and $\overset{\wedge }{\text{Α}}$ will not be equal to Λ and Α respectively, because SVD is not unique. Any non-singular orthogonal matrix [17] $Q\in R^{3K\times 3K}$ can be inserted between $\overset{\wedge }{\text{Λ}}\overset{\wedge }{\text{Α}}$, and get a new valid decomposition $W=\overset{\wedge }{\Lambda }\overset{\wedge }{\text{Α}}=\overset{\wedge }{\Lambda }QQ^{-1}\overset{\wedge }{\text{Α}}=\Lambda \text{Α}$. The matrix Q is called the rectification matrix. 

According to reference [6], instead of computing the whole matrix Q, only three columns of Q are sufficient to rectify $\overset{\wedge }{\Lambda }$ and $\overset{\wedge }{Α}$. After defining the first, $K+1^{st}$ and $2K+1^{st}$ columns of the rectification matrix Q as $Q_{k}$, we can get the R:

(10) $$\overset{\wedge }{\Lambda }Q_{k}=\left[\begin{array}{c} Q_{11}R_{1}\\ \vdots \\ Q_{M1}R_{M} \end{array}\right]$$

(11) $${\overset{\wedge }{\Lambda }}_{2i-1:2i}Q_{k}{\left({\overset{\wedge }{\Lambda }}_{2i-1:2i}Q_{k}\right)}^{T}={\overset{\wedge }{\Lambda }}_{2i-1:2i}Q_{k}Q_{k}^{T}{\overset{\wedge }{\Lambda^{T}}}_{2i-1:2i}={\text{θ}}_{i,1}^{2}I_{2\times 2},i=1,2,...,M$$

where ${\overset{\wedge }{\Lambda }}_{2i-1:2i}\in R^{2\times 3K}$ denotes the two rows of matrix $\overset{\wedge }{\Lambda }$ at positions between 2i−1 and 2i. 

Due to the inherent ambiguity of the orthogonal constraint, Xiao *et al.* [18] found that the above method couldn’t obtain a unique solution of the rectification matrix Q. However, Akhter *et al.* [14] showed that the inherent ambiguity did not necessarily lead to a fuzzy shape. Experimental results proved that only using the constraint can also recover the unique structure *S*.

The rectification matrix $Q_{k}$ can be estimated precisely by using the trace minimization constraint of the $Q_{k}Q_{k}^{T}$. Once matrix $Q_{k}$ has been computed, the matrix R can be estimated by using a nonlinear minimization routine. 

According to Equation (8), the structure matrix S can be calculated by the pseudo-inverse method. Because $(\Lambda^{T}\Lambda {)}^{-1}\Lambda^{T}\Lambda =E$, W=ΛA, coefficient matrix A is calculated as follows:

(12) $$A={\left(\Lambda^{T}\Lambda \right)}^{-1}\Lambda^{T}W$$

Then the structure matrix is calculated by the equation $S=\text{Θ}A=\text{Θ}{\left(\Lambda^{T}\Lambda \right)}^{-1}\Lambda^{T}W$. The S is set as the iterative initial value of the APG algorithm.

## 4. The Optimization of Matrix *S* Using the APG Algorithm

### 4.1. The Trace-Minimization Problem

The goal of this paper is to solve the structure matrix S through the equation W=RS, where the measurement matrix W is known, and the rotation matrix R is calculated. Because S=ΘA, the rank of the matrix should meet the requirement of the low-order linear model:

(13) rank(S)≤min{rank(Θ),rank(A)}≤3K

The size of DCT basis *K* is a small constant, so the structure matrix *S* is a low-rank matrix. Then the low-rank condition is relaxed to a rank-minimization problem [19,20]. Now the structure matrix S will be a solution to the rank minimization problem as follows:

(14) $$\begin{array}{l} \min rank(S),s.t\\ W=RS \end{array}$$

according to Dai *et al.* [21], because the rank-function itself is not very numerically stable and rank-minimization is an NP-hard problem in general. Relaxing the above rank-minimization to a nuclear-norm minimization form in an effective way [22,23], that is min ${\text{||S||}}_{\text{*}}$. In principle, the nuclear-norm minimization may be solved by a standard SDP solver [15]. In this study, the size of S is 3M×N. However, when the size is large, the SDP technique cannot work well.

### 4.2. The Application of the APG Algorithm

Many efficient convex optimization algorithms could be used to solve the problem. In this paper, an effective iterative algorithm, the APG algorithm [24,25], is proposed to optimize the non-rigid structure estimation from monocular vision. According to this algorithm, a closed form solution of the following Equation (15) can be obtained. In Equation (8), W is a measurement matrix of the signal S, which was obtained by using the calculated matrix R. The above minimization Equation (14) can be rewritten in Lagrangian form as follows:

(15) $$min\;\frac{1}{2}\left|\left|W-RS\right|\right|{\;}_{F}^{2}+\mu \left|\left|S\right|\right|{\;}_{*}$$

where μ>0 is a given parameter, and set $f\left(S\right)=\frac{1}{2}\left|\left|W-RS\right|\right|{\;}_{F}^{2}$, $P\left(S\right)=\mu \left|\left|S\right|\right|{\;}_{*}$, F(S)=f(S)+P(S)*.*

Here, the stopping condition of the APG algorithm is defined as following:

(16) $$\frac{{\text{||S}}_{\text{K}+\text{1}}{\text{-S}}_{\text{K}}{\text{||}}_{F}}{L_{f}\max\{1,||{\text{S}}_{\text{K}}|{|}_{F}\}}\leq tol$$

where *tol* is a moderately small positive number, since when $S_{K}$ gets close to an optimal solution S, the distance between $S_{K}$ and $S_{K+1}$ should become very small. If *tol* is too large, the non-rigid structure may not be calculated accurately, if *tol* is too small, the running time will be too long, so we should choose a suitable *tol*.

$L_{f}$ is the *Lipschitz* constant of ∇f:

(17) $$\text{||}\nabla f(S_{1})-\nabla f(S_{2})\text{||}\leq {\text{L}}_{f}||S_{1}-S_{2}||$$

where the *Lipschitz* constant L*f* is simply the square of the operator norm of the linear map: S→W. 

Instead of directly minimizing F(S), the APG method minimizes a sequence of separable quadratic approximation to F(S), denoted as $Q(S,S_{2})$, formed at specially chosen points $S_{2}$:

(18) $$Q(S,S_{2})=f(S_{2})+\langle \nabla f(S_{2}),S-S_{2}\rangle +\frac{\tau }{2}||S-S_{2}{||}_{F}^{2}+P(S)$$

Because:

(19) $$\arg\underset{X}{\min}Q(S,S_{2})=\arg\underset{X}{\min}\frac{L_{f}}{2}||S-S_{2}+\frac{1}{L_{f}}\nabla f(S_{2}){||}_{F}^{2}+P(S)$$

and then the iterative formula is shown as follows:

(20) $$\left\{ \begin{array}{c} Y_{k}=S_{k}+\frac{t_{k-1}-1}{t_{k}}(S_{k}-S_{k-1})\\ S_{k+1}=D_{\frac{\mu }{L_{f}}}(Y_{k}-\frac{1}{L_{f}}\nabla f(S_{k})) \end{array}\right.$$

The detailed steps of the APG algorithm are summarized in Algorithm 1.

**Algorithm 1.** The steps of the APG algorithm.Step 1. Initialization: Given μ>0, $S_{1}=S_{\text{0}}=S$, $t_{1}=t_{0}\in [1,+\infty )$, *K*=1, 2, 3...Step 2. While not converged doStep 3. $Y_{K}=S_{K}+\frac{t_{K-1}-1}{t_{k}}(S_{K}-S_{K-1})$Step 4. $G_{K}=Y_{K}-\frac{1}{L_{f}}R^{*}(RS_{K}-W)$Step 5. $S_{L_{f}}(G_{K})=UD\text{iag}({\left(\sigma -\mu /L_{f}\right)}_{+})V^{T}$Step 6. $S_{K+1}=S_{L_{f}}(G_{K})$, $t_{K+1}=\frac{1+\sqrt{1+4t_{K}^{2}}}{2}$Step 7. End while.Step 8. $S_{K+1}$, namely the reconstructed structure matrix S.

In the Algorithm 1, factorize G with SVD method, $G=U\Sigma V^{T}$, Σ=Diag(σ).

## 5. Experiment Results

### 5.1. The Yoga Sequence Experiment 

**Figure 1:**
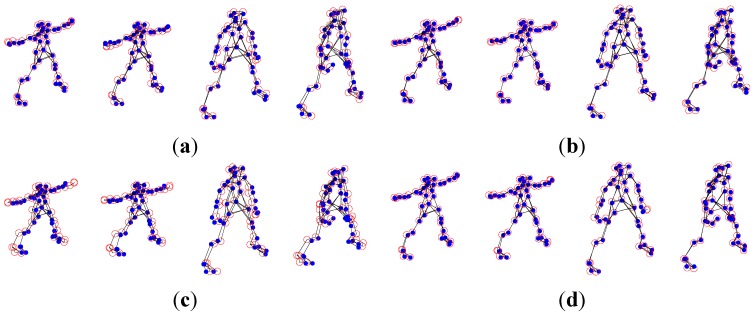
Reconstruction of the yoga sequence using the PTA method with four different *K* values, (**a**) *K* = 4; (**b**) *K* = 7; (**c**) *K* = 9; and (**d**) *K* = 11.

**Figure 2:**
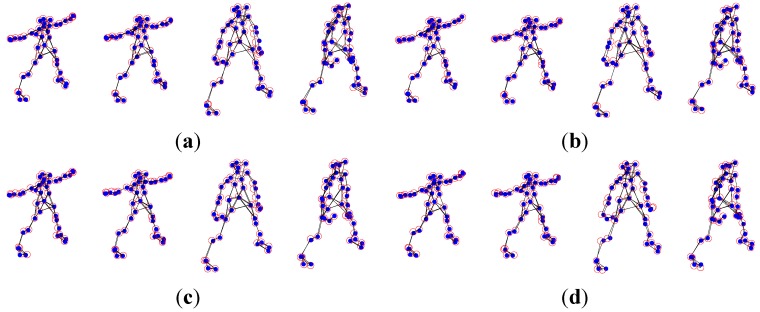
Reconstruction of the yoga sequence using the APG method with four different *K* values, (**a**) *K* = 4; (**b**) *K* = 7; (**c**) *K* = 9; and (**d**) *K* = 11.

The experimental dataset consists of a 307-frame sequence of a human practicing yoga, which comes from http://cvlab.lums.edu.pk/non-rigid structure estimation. The database is observed by a perspective camera orbiting the subject on a horizontal plane at a speed of 5° per frame. The reconstruction performances of Akhter *et al.*’s PTA approach and the APG approach are presented in the following Figure 1 and Figure 2, respectively. In the figures, the blue dots are the ground truth 3D points, and the red circles show the recovered points.

As shown in Figure 1 and Figure 2, in general, the APG method reconstruction is better than that of PTA algorithm. Especially, the reconstruction precision of the APG method is increased significantly when *K* = 9.

**Figure 3:**
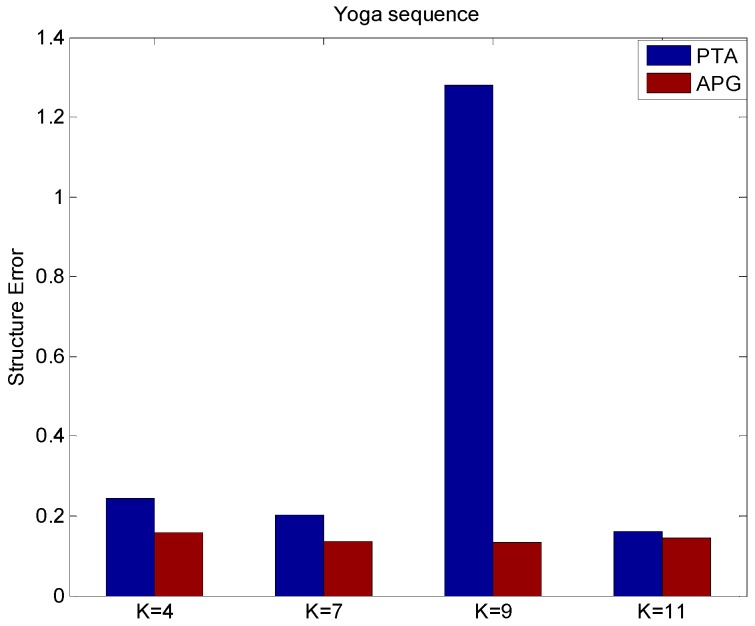
The structure error of different values of *K* by using PTA and APG algorithms.

From Figure 3, it can be found that APG method can improve the reconstruction quality with less reconstruction structure errors than the PTA method with different values of *K*. 

**Figure 4:**
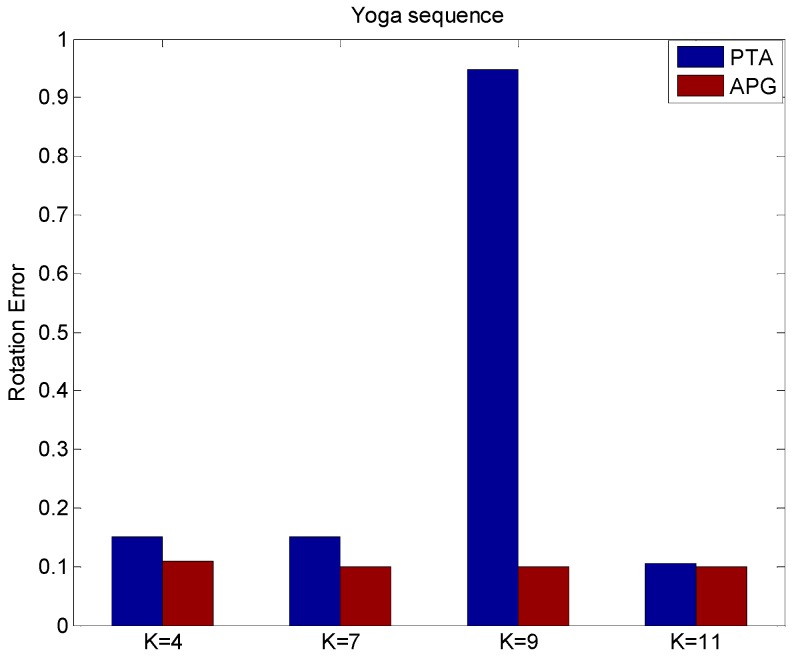
The rotation error of different values of *K* by using PTA and APG algorithms.

From Figure 4, it can be found that APG method can improve the reconstruction quality with less reconstruction rotation errors than the PTA method with different values of *K*. From the above figures, the proposed method performs effectively, and the reconstruction accuracy of non-rigid structure estimation from monocular vision is improved effectively. The reconstruction results on the real yoga sequence images indicate that not only the structure reconstruction, but also the rotation reconstruction is obviously improved by using the APG algorithm. 

### 5.2. The Pickup Sequence Experiment

In addition, another experimental dataset is proposed to test the proposed APG method for non-rigid structure estimation. The experimental dataset consists of a 357-frames human pickup sequence, which come from http://cvlab.lums.edu.pk/non-rigid structure estimation. The database is observed by a perspective camera orbiting the subject on a horizontal plane at a speed of 5° per frame. In this paper, the comparison of non-rigid structure estimation from monocular vision between the proposed APG algorithm and PTA algorithm is given, which are presented in terms of the reconstruction result figures and reconstruction error curves with different *K* values. In the following figures, the blue dots are the ground truth 3D points, and the red circles show the recovered points.

**Figure 5:**
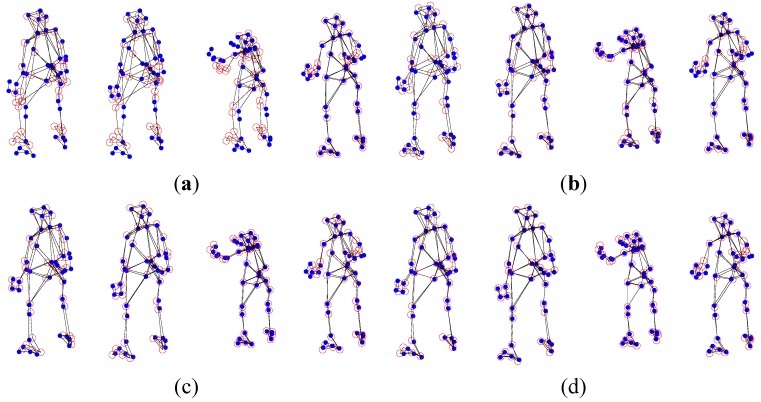
PTA reconstruction of the pickup sequence with four different *K* values, (**a**) *K* = 3; (**b**) *K* = 7; (**c**) *K* = 10; and (**d**) *K* = 12.

**Figure 6:**
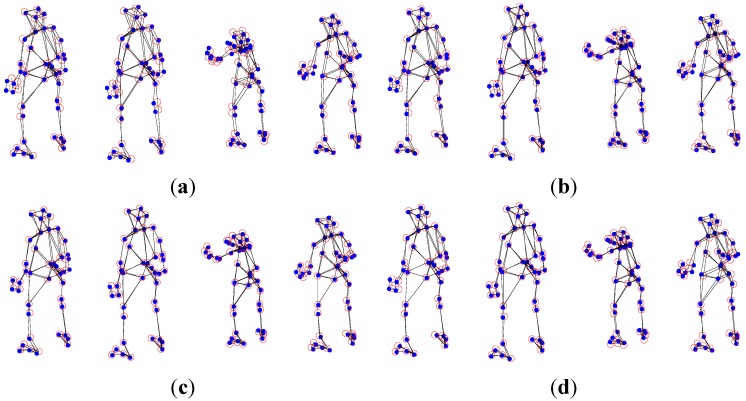
APG method reconstruction of the pickup sequence with four different *K* values, (**a**) *K* = 3; (**b**) *K* = 7; (**c**) *K* = 10; and (**d**) *K* = 12.

From Figure 5 and Figure 6, in general, the APG method reconstruction is better than the PTA algorithm one. When *K* = 3, the reconstruction precision of the APG method is increased significantly.

**Figure 7:**
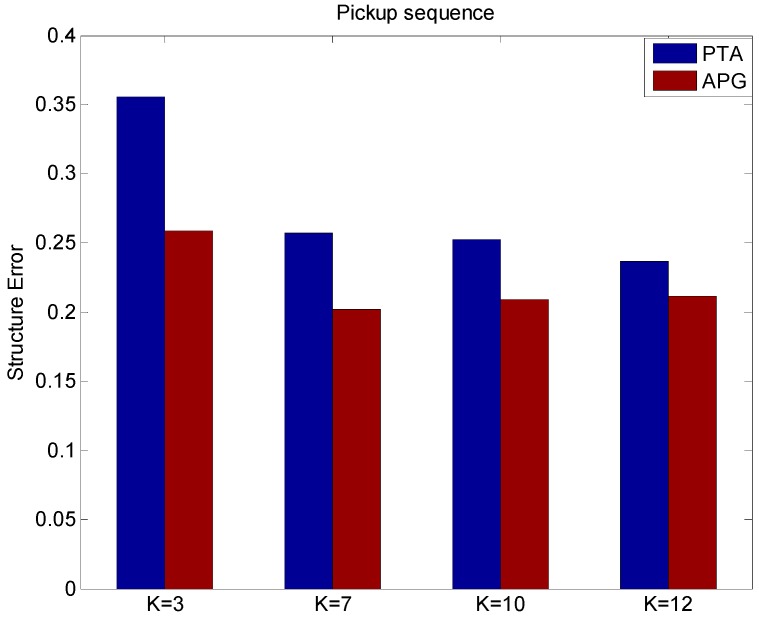
The structure error of different values of *K* using the PTA and APG algorithms.

From Figure 7, it can be found that APG method can improve the reconstruction quality with less reconstruction structure errors than the PTA method with different values of *K*.

**Figure 8:**
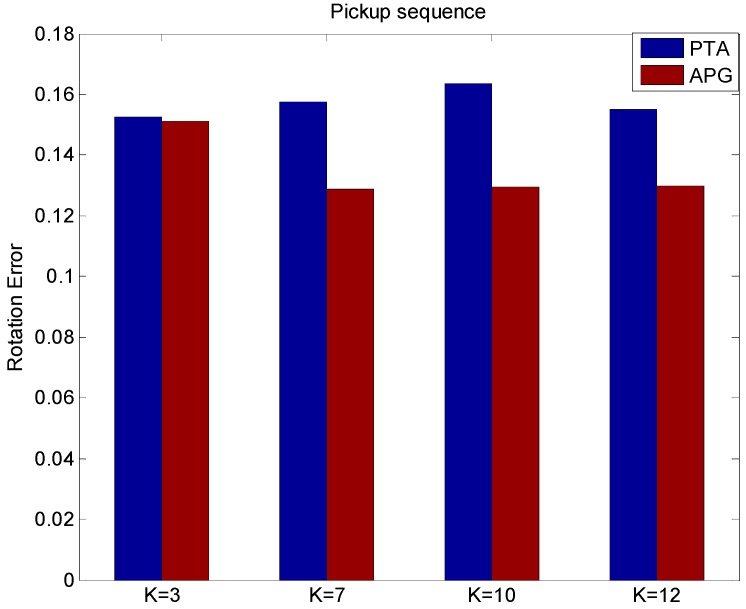
The rotation error of different values of *K* using the PTA and APG algorithms.

From Figure 8, it can be found that APG method can improve the reconstruction quality with less reconstruction rotation errors than the PTA method with different values of *K*. From Figure 7 and Figure 8, we can see the proposed APG method runs reliably, and the accuracy of non-rigid structure estimation is effectively improved. The reconstruction results on the real pickup image sequences indicate that the APG method outperforms the PTA method in terms of reconstruction accuracy of non-rigid structure estimation.

### 5.3. The Comparison of APG Method and Block Matrix Method

The comparisons of reconstruction results between the APG algorithm and Block Matrix Method (the method of Dai *et al.* [20]) on the shark sequence are presented in the following figure. The shark sequence contains 240 frames and 91 features.

**Figure 9:**
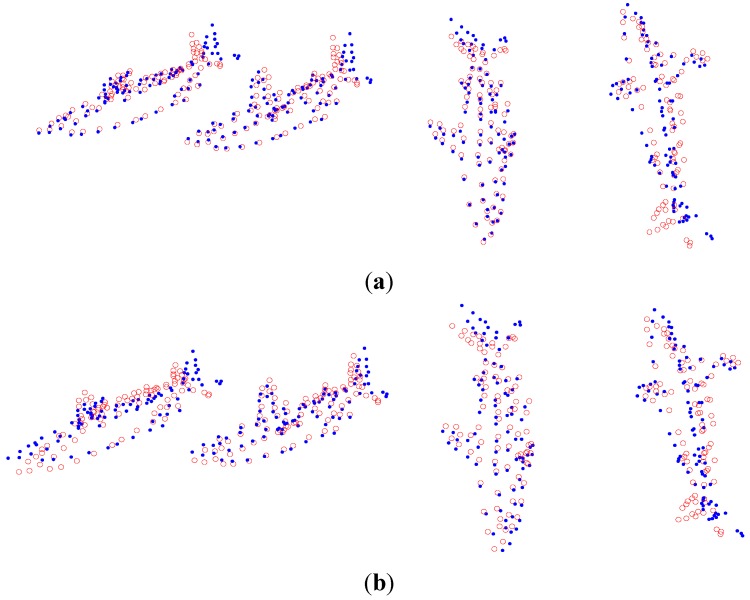
3D reconstruction results on the shark sequence. (**a**) Reconstruction results using the APG method, where the mean structure error is 0.204; (**b**) Reconstruction results using the Block Matrix Method, where the mean structure error is 0.242.

**Figure 10:**
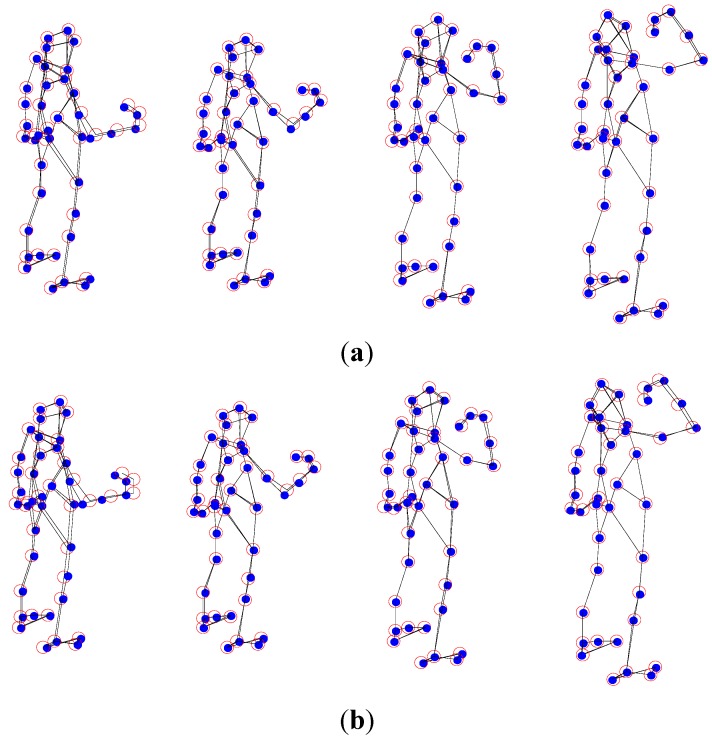
3D reconstruction results on the drink sequence. (**a**) Reconstruction results using the APG method, where the mean structure error is 0.017; (**b**) Reconstruction results using the Block Matrix Method, where the mean structure error is 0.019.

The comparison of reconstruction errors between the APG algorithm and Block Matrix Method is provided in Table 1, where the number in brackets is the best value of *K*, which is chosen by exhaustively trying out different numeric values between 2 and 13. In our paper, the best value of K is confirmed by the tracked positions of a sequence of non-rigid shapes by using the rank analysis method [26]. As can be seen in Figure 9 and Figure 10 and Table 1, the proposed APG method outperforms the Block Matrix Method when the experiment involves the shark and drink sequences. When the experiment is about other sequences, the APG method is not good as the Block Matrix Method, but the difference is not obvious.

All the structure error mentioned in our paper refers to the mean error of each frame, we call it mean structure error ($e_{S}\left(t\right)$). The computational formula is as follows:

(21) $$e_{S}\left(t\right)=\frac{1}{\sigma N}\sum_{j=1}^{N}e_{tj},\text{σ}=\frac{1}{3T}\sum_{t=1}^{T}\left(\sigma_{tx}+\sigma_{ty}+\sigma_{tz}\right)$$

(22) $$e_{tj}=\sqrt{L_{tjx}^{2}+L_{tjy}^{2}+L_{tjz}^{2}}$$

(23) $$L_{tjx}=\left|S_{r}\left(3t-2,j\right)-S_{0}\left(3t-2,j\right)\right|$$

(24) $$L_{tjy}=\left|S_{r}\left(3t-1,j\right)-S_{0}\left(3t-1,j\right)\right|$$

(25) $$L_{tjz}=\left|S_{r}\left(3t,j\right)-S_{0}\left(3t,j\right)\right|$$

where  j=1,2,…,N, $\sigma_{tx},\;\;\sigma_{ty}$ and $\sigma_{tz}\;$ are respectively the standard deviation of the point X,Y,Z coordinates of the *t*-th frame corresponding to the 3D structure. $e_{tj}$ represents the reconstruction error of the j-th 3D point of the *t*-th frame. $S_{r}\;$ is the structure matrix we reconstructed, while $S_{0}$ is the actual structure matrix.

**Table 1:** The reconstruction error by using APG method and Block Matrix Method.

Database	Shark	Drink	Yoga	Dance	Pickup
Block Matrix Method	0.242(3)	0.019(4)	0.125(9)	0.171(10)	0.138(7)
APG method	0.204(2)	0.017(13)	0.135(9)	0.231(5)	0.202(7)

### 5.4. The Comparison of the APG Method and Existing Methods

**Figure 11:**
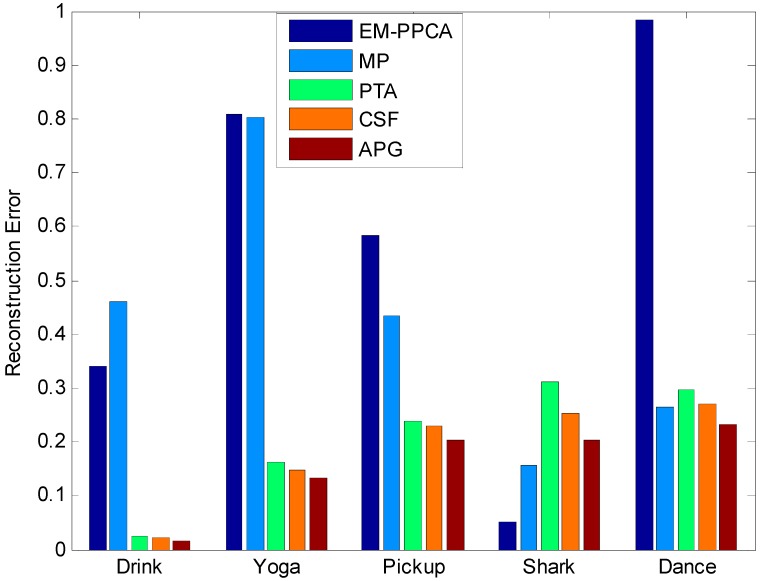
The structure error of five different sequences by using different methods.

Moreover, the comparison of reconstruction error between the APG algorithm and the existing EM-PPCA [17], MP [27], the PTA method [6], and CSF [9] are also provided in Figure 11 and Table 2. 

The abscissa of Figure 11 shows five sequences: drink, yoga, pickup, shark and dance, respectively. Its ordinate indicates the structure errors using five different methods. It can be found that the reconstruction results using the APG method are obviously better than those obtained with the other methods. 

From Figure 11 and Table 2, the reconstruction results on the most real image sequences indicate that reconstruction accuracy is significantly improved by the APG method, which obviously reduces the structure errors. The APG method can further optimize the structures calculated by the PTA method, which is proved to be an effective approach for non-rigid structure estimation from monocular vision. However, for dramatic movement image sequence, the proposed approach cannot improve the accuracy of the non-rigid structure estimation effectively, such as in the shark sequence.

In our algorithm, the PTA method is first used to calculate the structure matrix, and then the APG method is used to optimize the structure matrix. In the total execution time of our algorithm (T), the percentage of the execution time of APG algorithm (t/T) is showedn in Table 3.

**Table 2:** APG method compared with other methods in terms of reconstruction error.

Database	Methods				
	EM-PPCA	MP	PTA	CSF	APG
Drink	0.339	0.460	0.025 (3)	0.022 (6)	0.017 (13)
Dance	0.984	0.264	0.296 (5)	0.271 (2)	0.231 (5)
Yoga	0.810	0.804	0.162 (11)	0.147 (7)	0.135 (9)
Shark	0.050	0.157	0.312 (2)	0.254 (2)	0.204 (2)
Pickup	0.582	0.433	0.237 (12)	0.230 (6)	0.202 (7)

**Table 3:** The percentage of the execution time of APG algorithm.

Time	Drink	Dance	Yoga	Shark	Pickup
*T*	30.073 s	1.9245 s	2.4637 s	0.8516 s	3.7898 s
*t/T*	4.99%	19.1%	3.12%	23.9%	2.62%

Table 3 shows that our APG method runs quickly, and can converge to the best solution in a little time.

## 6. Conclusions

In this paper, the APG algorithm is proposed to solve the trace-minimization problem of the structure matrix. The initial value of the APG algorithm can be calculated by using the PTA method. The proposed APG method can further improve the structure performance and converge to the optimal solution. Above experimental databases are applied to test the proposed APG method for non-rigid structure estimation from monocular vision. The experimental results show that the proposed method can improve the reconstruction quality of non-rigid structure estimation with less reconstruction error than the PTA method. The APG algorithm can also converge to the best solution quickly, so the time consumption of the proposed method is near that of the PTA method. 

However, the APG method is not available for reconstructing dramatic movement sequences, so in the future, the APG method will be improved to make it suitable for dramatic movement. Moreover, the selection of the initial value plays an important role in the reconstruction efficiency of the proposed algorithm, and how to select the best initial value is another future work. The structure matrix *S* can also act as a solution to the rank minimization problem, so in the future, some optimization algorithms, such as the Singular Value Thresholding (SVT) algorithm, will be considered to further reduce the reconstruction error of non-rigid structure estimation from monocular vision.
